# Green-sensitive opsin is the photoreceptor for photic entrainment of an insect circadian clock

**DOI:** 10.1186/s40851-015-0011-6

**Published:** 2015-02-26

**Authors:** Sayaka Komada, Yuichi Kamae, Mitsumasa Koyanagi, Kousuke Tatewaki, Ehab Hassaneen, ASM Saifullah, Taishi Yoshii, Akihisa Terakita, Kenji Tomioka

**Affiliations:** Graduate School of Natural Science and Technology, Okayama University, Okayama, 700-8530 Japan; Bangladesh Atomic Energy Commission, Dhaka, 1212 Bangladesh; Graduate School of Science, Osaka City University, Osaka, 558-8585 Japan

**Keywords:** Circadian clock, Compound eye, Cricket, Entrainment, Opsin, RNAi

## Abstract

**Introduction:**

Entrainment to light cycle is a prerequisite for circadian rhythms to set daily physiological events to occur at an appropriate time of day. In hemimetabolous insects, the photoreceptor molecule for photic entrainment is still unknown. Since the compound eyes are the only circadian photoreceptor in the cricket *Gryllus bimaculatus*, we have investigated the role of three opsin genes expressed there, *opsin-Ultraviolet* (*opUV*), *opsin-Blue* (*opB*), and *opsin-Long Wave* (*opLW)* encoding a green-sensitive opsin in photic entrainment.

**Results:**

A daily rhythm was detected in mRNA expressions of *opB* and *opLW* but not of *opUV* gene. When photic entrainment of circadian locomotor rhythms was tested after injection of double-stranded RNA (dsRNA) of three opsin genes, no noticeable effects were found in *opUV* RNAi and *opB* RNAi crickets. In *opLW* RNAi crickets, however, some crickets lost photic entrainability and the remaining crickets re-entrained with significantly longer transient cycles to a phase-advanced light–dark cycle as compared to control crickets. Crickets often lost entrainability when treated doubly with dsRNAs of two opsin genes including *opLW.*

**Conclusion:**

These results show that green-sensitive OpLW is the major circadian photoreceptor molecule for photic entrainment of locomotor rhythms in the cricket *G. bimaculatus.* Our finding will lead to further investigation of the photic entrainment mechanism at molecular and cellular levels, which still remains largely unknown.

**Electronic supplementary material:**

The online version of this article (doi:10.1186/s40851-015-0011-6) contains supplementary material, which is available to authorized users.

## Introduction

Circadian rhythms are about 24-hour oscillations observed in various physiological functions of a wide variety of organisms. The rhythms are controlled by an endogenous mechanism called the circadian clock. The most important role of the clock is to set physiological functions to peak at an appropriate time of day. Therefore its synchronization to the environmental daily cycle is a prerequisite and the clock uses light as a primary cue (zeitgeber) for this purpose. In insects, two pathways have been identified. One involves extraretinal photoreceptors that are confined within or in the vicinity of clock cells. In *Drosophila melanogaster* and the monarch butterfly (*Danaus plexippus*), a blue light receptor, CRYPTOCHROME (CRY), that is expressed in a subset of cerebral clock neurons, mediates the photic information, leading to a light-dependent degradation of TIMELESS protein [1-6]. In honey bees (*Apis mellifera*), another photoreceptor molecule, pteropsin [7], might be involved in this mechanism. The other external pathway plays an additional role in photic entrainment in *D. melanogaster.* The compound eye contributes to some extent to photic entrainment [8,9]; a subset of cerebral pacemaker neurons also respond to nocturnal dim light through retinal input [10]. In hemimetabolous insects, including crickets and cockroaches, the compound eye is the major photoreceptor and the light information perceived by the eye is conducted through neural pathways to the clock, which is located in the optic lobe [11,12]. The cerebral photoreceptors may exist but play only minor roles [13,14].

The entrainment through retinal photoreceptors resembles that known for the mammalian circadian clock that resides in the suprachiasmatic nucleus (SCN). The major photoreceptor, melanopsin, is expressed in a fraction of retinal ganglion cells of which axons project to the SCN through the retino-hypothalamic tract [15]. In the SCN their neurotransmitters, glutamate and PACAP, reset the cellular clock machinery that consists of so-called clock genes [16]. Similar process might be involved in circadian entrainment in hemimetabolous insects. Although investigation of action spectra of photic entrainment of locomotor rhythm showed that long wavelength light is most effective in cockroaches [17], the photoreceptor molecule and their role have not been clarified yet.

In the present study, cDNAs of three visual opsin genes, *opsin-Ultraviolet* (*opUV*), *opsin-Blue* (*opB*), and *opsin-Long Wave* (*opLW*) were obtained by molecular cloning in the cricket *Gryllus bimaculatus*, and their physiological roles in entrainment of circadian rhythm were analyzed. Measurement of the mRNA levels revealed that in adult male crickets, *opB* and *opLW* were rhythmically expressed but *opUV* was not. Analysis of the role of the opsin genes in the photic entrainment of the locomotor rhythm showed that most of the *opLW* RNAi crickets lost entrainability or showed weakened entrainability to light dark cycles. It is thus suggested that OpLW, a green sensitive opsin, is the major photoreceptor molecule involved in the photic entrainment of the cricket’s circadian clock.

## Materials and methods

### Animals

Adult male crickets, *Gryllus bimaculatus*, were used. They were obtained from a laboratory colony maintained under standard environmental conditions with a light–dark cycle of 12 h light to 12 h dark (LD12:12) (light: 06:00–18:00 h; Japanese standard time) at a constant temperature of 25 ± 0.5°C. They were fed a rodent diet, CA-1 (Clea Japan, Tokyo) and water.

### Cloning of *opsin* genes

Total RNA was extracted from the compound eyes and used for reverse transcription to synthesize first strand cDNA, using Superscript First-Strand Synthesis SuperMix for q-PCR (Invitrogen, Carlsbad, California) or SMART mRNA Amplification Kit (Clontech, Mountain View, California). With the obtained cDNA as a template and primers designed for each gene (Table 1), PCR was performed with a condition of 30 sec for denaturation at 95°C, 30 sec for primer annealing at 55–62°C, 0.5-2 min for extension at 72°C for 35 cycles with Ex taq DNA polymerase (Takara Bio., Ohtsu, Japan) or Blend taq-plus (Toyobo, Osaka, Japan). The purified fragment was cloned into T-Vector pMD20 vector (Takara) and sequenced with BigDye Terminator v3.1 Cycle Sequencing Kit (Applied Biosystems, Foster City, California). Sequences were analyzed by Genetyx version 6 (Genetic Information Processing Software, Tokyo, Japan) and BioEdit version 7.1.3.0 (Biological Sequence Alignment Editor, Ibis Therapeutic, Carlsbad, California). We got cDNAs of 879 bp, 613 bp, and 837 bp for *opUV*, *opB*, and *opLW*, respectively. We then performed 5′ and 3′ RACEs to obtain whole (*opUV*, *opB*) or nearly whole (*opLW*) cDNA sequences. The method was described elsewhere [18].

**Table 1:** **Primers for cloning and RNAi of opsin genes, and for real-time PCR of opsin and** ***rpl18a*** **genes**

**Gene**	**Forward (5′> > 3′)**	**Reverse (5′> > 3′)**
For cloning		
*Opsin-UV*	GAGTTAATCCACATCCCGGA	AGCTACAGCTTTGCAAGTGC
*Opsin-Blue*	TGCTCGACCTGCTAATGATG	ATTGCCACAGTAGCGTAGGG
*Opsin-LW*	CCGCTCTGGCACGGCCTCCTG	GTAGACGGCGTTGGCCTTGGCA
For dsRNA synthesis		
*Opsin-UV*	GCATGCATTGCCTATGACAG	TGCCAAATGCACCAATTAGA
*Opsin-Blue*	TGGTATTGGTTCTGCCATCA	ATTGCCACAGTAGCGTAGGG
*Opsin-LW*	GCGTGCTGGGAGTGATCT	GCCACGTCTTGGTCAGGTAG
For real time PCR		
*Opsin-UV*	ACATTCCCCGAGCCAGA	ACCAGTCCATTTCCCACGA
*Opsin-Blue*	CTTTGCTCGACCTGCTAATGA	CACAGCCTACTTCCCAACCAA
*Opsin-LW*	CGCTCCTACATCCTCGTCTACTC	CGTTCATCTTCTTGGCTTGCT
*rpl18a*	GCTCCGGATTACATCGTTGC	GCCAAATGCCGAAGTTCTTG

### RNAi

Double-stranded RNAs (dsRNA) for *opUV, opB, opLW* and *DsRed2* were synthesized according to the method described by Moriyama et al. [19] using MEGAscript1 High Yield Transcription Kit (Ambion, St. Austin, Texas). *DsRed2* was derived from a coral species (*Discosoma sp.*) and used as a negative control [19]. A DNA fragment of each gene was amplified with primer containing the T7 or T3 promoter (Table 1). With these DNA fragments, RNAs were synthesized with T7 or T3 RNA polymerases. The same amounts of sense and antisense RNAs were mixed, denatured for 5 min at 95°C, and annealed by a gradual cool down to room temperature. After ethanol precipitation, the obtained dsRNA was suspended in Ultra Pure Water (Invitrogen) and the concentration was adjusted to 20 μM. The dsRNA solutions were stored at –80°C until use. A 303.5 nL of 20 μM dsRNA solution was injected into both right and left compound eyes with the nanoliter injector (WPI, Sarasota, Florida). In combined RNAi, a 605 nL mixture of dsRNA of two genes, i.e., ds*opB* and ds*opLW*, ds*opUV* and ds*opLW,* or ds*opUV* and ds*opB*, were injected. For recording locomotor activity, the treated crickets were housed in the activity chamber in a few days after the dsRNA injection. For measurement of mRNA levels, the crickets were used one week after the dsRNA injection.

### Measurement of mRNA levels

Quantitative real-time RT-PCR (qPCR) was used to measure mRNA levels. Total RNA was extracted and purified with TRIzol Reagent (Invitrogen) from a compound eye of adults. To remove contaminating genomic DNA, total RNA was treated with DNase I. About 500 ng of total RNA of each sample was reverse transcribed with random 6mers using Primescript RT reagent kit (Takara).

The qPCR was performed by Mx3000P Real-Time PCR System (Stratagene, La Jolla, California) using FastStart Universal SYBR Green Master (Roche, Tokyo, Japan) including SYBR Green with the gene specific primers (Table 1). The results were analyzed using software associated with the instrument. The values were normalized with the values of *Gb’rpl18a* (GenBank/EMBL/DDBJ Accession No. DC448653), a housekeeping gene, at each time point. Existence of daily changes in expression of opsin genes and effects of dsRNA treatment on mRNA expression levels were examined by one-way ANOVA.

### Behavioral analysis

Locomotor activity of individual animals was recorded with an activity chamber made of transparent plastic box (17.6 × 8.7 × 4.4 cm) with a rocking substratum as described by Moriyama et al. [19]. In brief, a magnetic reed switch sensed rocking movement of the substratum caused by a moving cricket. The number of rocking was recorded every 6 min by a computerized system. Water and food were provided *ad libitum*.

The activity chambers were placed in an incubator in which temperature was kept at 25 ± 0.5°C. Desired lighting regimens were given by a white fluorescent lamp controlled by an electric timer, and the photon flux density was 1.7-17 μmol/m^2^/s, varying with the proximity to the lamp. Recording was performed under the standard LD cycle (LD 12:12, light: 06:00–18:00) for about one week, then LD cycle was advanced or delayed by 6 h. The raw data were displayed as conventional double-plotted actograms to judge activity patterns using ActogramJ [20]. Synchronization to the delayed or advanced light cycle was judged to be completed when the onset of nocturnal activity peak matched the light-off. The number of transient cycles was defined as the days necessary for re-entrainment after the phase shift including the day on which the shift was made. One-way ANOVA was used for statistical examination of the effects of RNAi of opsin genes on the number of transient cycles.

## Results

### Daily expression of the *opsin* genes

By molecular cloning we have obtained cDNAs of three opsin genes, i.e., *opUV* (GenBank/EMBL/DDBJ Accession No. LC004295), *opB* (LC004296), and *opLW* (LC004297), encoding product proteins of 377 aa, 379 aa, and 377 aa, respectively. The deduced amino acid sequences encoded by these opsin genes were identical to those reported by Henze et al. [21] but there were some nucleotide substitutions in each of the opsin genes. *In situ* hybridization of opsin mRNA revealed that *opLW* is expressed over almost the whole compound eye except for the dorsal rim area (DRA), *opUV* in a single proximally located cell in every ommatidium over almost the whole compound eye except for a ventral region, and *opB* expressed most abundantly in DRA and replaced with *opUV* in the ventral region (Additional file 1: Figure S1). The expression profile was consistent with that reported by Henze et al. [21] and similar to that in the cricket *Modicogryllus siamensis* [18]. The *opUV*, *opB*, and *opLW* are probably expressed in the retinula cells with a peak spectral sensitivity at 332 nm (UV), 445 nm (blue), and 515 nm (green), respectively, that were reported by Zufall et al. [22].

**Figure 1:**
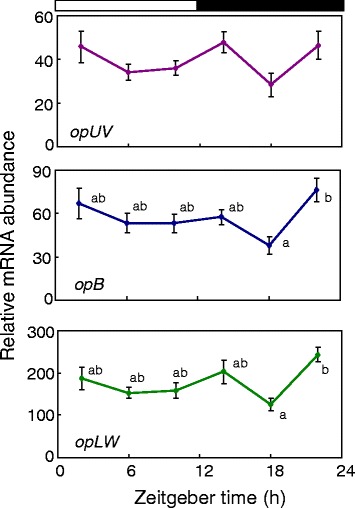
**Daily expression profiles of the** ***opUV*** **,** ***opB*** **, and** ***opLW*** **mRNAs in the compound eye of adult crickets.** The abundance of mRNAs was measured by quantitative real-time RT-PCR and expressed as relative values to the abundance of *rpl18a* mRNA that was used as an internal reference. The values are average of 8 compound eyes. The vertical lines indicate S.E.M. White and black bars indicate light and dark phase, respectively. A significant decrease occurred in the midnight, followed by a significant increase in the late night in *opB* and *opLW* (P < 0.05, ANOVA followed by Tukey-test). Values labelled with different letters significantly differ from each other.

To examine whether transcripts of the opsin genes oscillated in a circadian manner, we measured mRNA levels of *opUV*, *opB,* and *opLW,* in the compound eye by qPCR (Figure 1). The samples were collected at 4 h intervals starting at 2 h after lights-on (ZT 2: ZT stands for zeitgeber time and ZT 0 corresponds to lights-on and ZT 12 to lights-off). The mRNA levels of the three opsin genes showed a slight reduction at midnight followed by an increase at late night and these changes were statistically significant for *opB* and *opLW* (P < 0.05, ANOVA followed by Tukey-test). However, no significant daily change was detected in *opUV* (P > 0.05, ANOVA).

### dsRNA suppressed mRNA levels of the opsin genes

**Figure 2:**
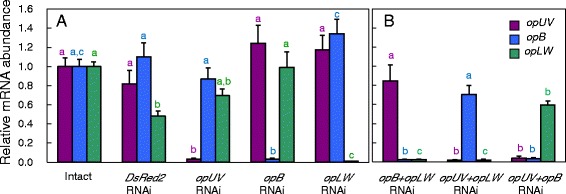
**Effects of single (A) or combined treatment (B) with dsRNA of** ***opUV*** **,** ***opB*** **, and** ***opLW*** **on mRNA levels of opsin genes in the adult cricket compound eye at ZT 6.** *DsRed2* was used as a negative control. The mRNA levels were measured by quantitative real-time RT-PCR. The abundance of *rpl18a* mRNA was used as an internal reference. The values are average of 8 compound eyes and expressed relative to those of intact crickets. The black vertical lines indicate S.E.M. One-way ANOVA was performed for each opsin genes with intact crickets, and those treated with *DsRed2* RNAi and single and double RNAi of opsin genes. Values labelled by different letters significantly differ each other (P < 0.05, ANOVA followed by Tukey-test). Purple, blue, and green letters are for *opUV*, *opB*, and *opLW*, respectively. The dsRNAs significantly suppressed respective opsin genes. *opLW* mRNAs were also suppressed in *opUV* and *opB* double dsRNA treatment **(B)**. For further explanations see text.

To examine whether RNAi of the opsin genes worked effectively in *G. bimaculatus*, levels of *opUV*, *opB,* and *opLW* mRNAs were measured by qPCR in the compound eye of adult male crickets treated with respective dsRNA. The crickets were collected at 12:00 (ZT 6). The mRNA levels of treated opsin genes were found to be significantly lower than those of intact crickets (P < 0.05, ANOVA followed by Tukey-test), suggesting that dsRNA of the opsin genes suppressed mRNA levels of respective opsin genes through RNAi (Figure 2A); The mRNA levels of *opUV*, *opB*, and *opLW* was 3.0%, 3.4%, and 1.2% of intact crickets, respectively. Slight changes were observed in other opsin mRNA levels but no significant reduction was found (P > 0.05, ANOVA followed by Tukey-test). In control crickets treated with dsRNA of *DsRed2*, a significant reduction by 45% was observed in *opLW*, but no significant reduction was observed in *opUV* and *opB*.

Similarly double-RNAi of two opsin genes effectively knocked down respective opsin genes (Figure 2B). The *opB* and *opLW* RNAi reduced respective mRNA levels to 2.2% and 2.3% (P < 0.05, ANOVA followed by Tukey-test), the *opUV* and *opLW* RNAi reduced to 1.6% and 1.9% (P < 0.05, ANOVA followed by Tukey-test), and the *opUV* and *opB* RNAi reduced to 4.0% and 3.3% (P < 0.05, ANOVA followed by Tukey-test). Although the *opUV* and *opB* RNAi treatments significantly reduced *opLW* (P < 0.05, ANOVA followed by Tukey-test), abundant *opLW* mRNAs were still expressed (Figure 2B).

### Effects of RNAi of the opsin genes on the photic entrainment

**Figure 3:**
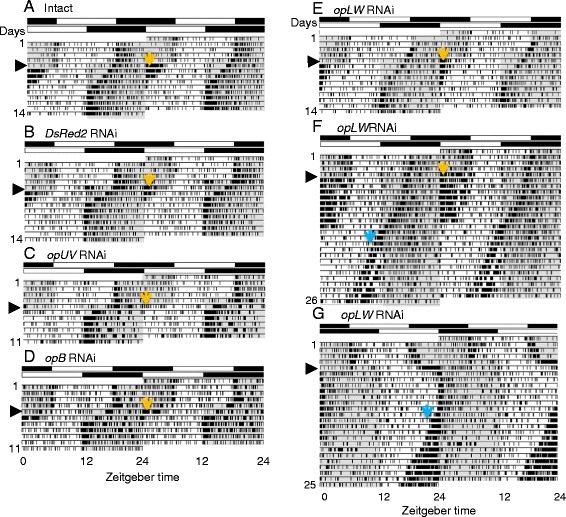
**Double plotted actograms of locomotor activity of intact (A),** ***DsRed2*** **RNAi (B),** ***opUV*** **RNAi (C),** ***opB*** **RNAi (D), and** ***opLW*** **RNAi crickets (E-G).** Light cycles were advanced **(A-F)** or delayed **(G)** by 6 h on the day indicated by an arrow head. White and black bars above actograms indicate light (white) and dark (black) phases. Shaded areas in actograms indicate dark phases. *DsRed2* **(B)**, *opUV* **(C)**, and *opB* **(D)** RNAi crickets showed re-entrainment to the advanced LD in almost similar time course to that of intact cricket **(A)**. *opLW* RNAi crickets showed a re-entrainment with longer transient cycles **(E)** or a free-running rhythm without re-entrainment **(F and G)**. Note all of the crickets showed intense activity bouts at lights-on after phase advance (orange arrow, **A**-**E**) or enhanced activity peak in the light phase (blue arrow, **F**, **G**) when the subjective night was exposed to light.

**Figure 4:**
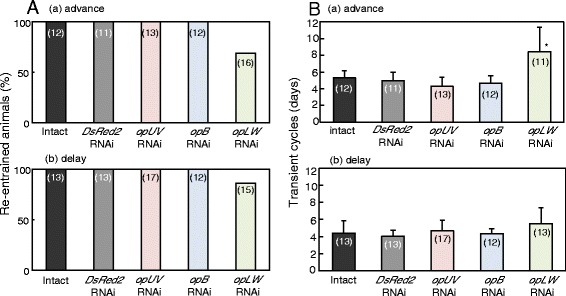
**The ratio of re-entrainment (A) and number of transient cycles (B) in intact crickets and those treated with ds** ***DsRed2*** **, ds** ***opUV*** **, ds** ***opB*** **or ds** ***opLW*** **in (a) 6 h phase advanced or (b) phase delayed conditions.** The vertical lines in B indicate S.D. The numbers in parenthesis indicate the number of animals used. Note that *opLW* RNAi disrupted re-entrainment in certain fraction of crickets **(A)** and significantly lengthened the transient cycles for advance shift compared with intact and *DsRed2* RNAi crickets (*, P < 0.05, ANOVA followed by Tukey-test) **(B)**.

To investigate whether the opsins are involved in the photic entrainment of crickets, re-entrainment to LD shifted by 6 h was examined in crickets treated with dsRNA of *opUV*, *opB,* or *opLW*. Crickets treated with *DsRed2* dsRNA were used as a negative control. Figure 3 exemplifies the locomotor activity records of crickets treated with ds*DsRed2*, ds*opUV*, ds*opB,* and ds*opLW*. All of the RNAi crickets treated with ds*DsRed2*, ds*opUV*, or ds*opB* re-entrained to the shifted LD cycles like intact crickets (Figures 3A-D and 4). After the LD phase advance, an intense activity often occurred at the beginning of the new light phase, which was a masking effect caused by unpredictable light exposure of the subjective night (Figure 3A, B, D). The numbers of transient cycles were 5.3 ± 0.9 (average ± S.D.) days, 5.0 ± 1.0 days, 4.3 ± 1.1 days, and 4.7 ± 0.9 days for intact, *DsRed2* RNAi, *opUV* RNAi and *opB* RNAi crickets, respectively, for advance, and 4.4 ± 1.4 days, 4.0 ± 0.7 days, 4.7 ± 1.2 days and 4.3 ± 0.6 days, respectively, for the delay shift (Figure 4). Some of the *opLW* RNAi crickets lost entrainability to free-run as exemplified in Figure 3F and G. Even in re-entrained animals longer transient cycles were necessary for resynchronization (Figures 3E and 4); 8.5 ± 2.9 days and 5.5 ± 1.9 days for advance and delay shifts, respectively, and the former was significantly longer than in intact crickets and negative controls treated with ds*DsRed2* (P < 0.05, ANOVA followed by Tukey-test). Even in *opLW* RNAi crickets, a masking effect of light was clearly observed at lights-on when the subjective night was exposed to light (Figure 3E). Interestingly, they also showed enhancement of nocturnal activity peak when the active phase occurred in the light phase (Figure 3F, G).

**Figure 5:**
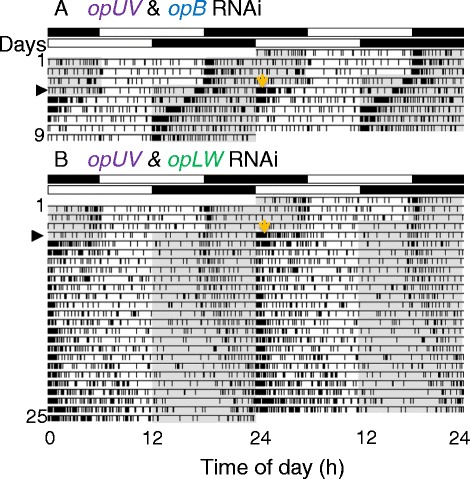
**Double plotted actograms showing locomotor activity of crickets doubly treated with ds** ***opUV*** **and ds** ***opB*** **(A) and ds** ***opUV*** **and ds** ***opLW*** **(B) that subjected to LD cycles advanced by 6 h on the day indicated by an arrow head.** White and black bars above actograms indicate light (white) and dark (black) phases. Shaded areas in actograms indicate dark phases. The *opUV* and *opB* double RNAi cricket (expressing mainly *opLW*) clearly synchronized to the shifted LD **(A)** while the *opUV* and *opLW* double RNAi cricket (expressing mainly *opB*) did not. Note that the crickets showed intense activity bouts at lights-on (orange arrow) after phase advance when the subjective night was exposed to light.

**Figure 6:**
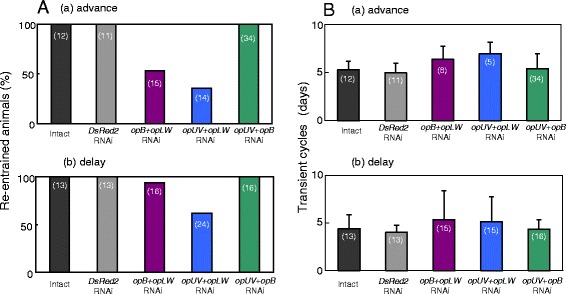
**The ratio of re-entrainment (A) and number of transient cycles (B) in intact and** ***DsRed2*** **RNAi crickets and those doubly treated with ds** ***opB*** **and ds** ***opLW*** **, ds** ***opUV*** **and ds** ***opLW,*** **or ds** ***opUV*** **and ds** ***opB*** **in (a) 6 h phase advanced or (b) phase delayed conditions.** The vertical lines in B indicate S.D. Note that treatment of double RNAi including ds*opLW* disrupted re-entrainment in certain fraction of crickets **(A)**, and slightly lengthened the transient cycles compared with intact and *DsRed2* RNAi crickets **(B)** but statistical significance was not detected by ANOVA.

To further investigate the role of each opsin, photic entrainment was examined in crickets treated with dsRNAs of two opsin genes, i.e., *opB* and *opLW*, *opUV* and *opLW*, and *opUV* and *opB*. All double RNAi crickets without ds*opLW* treatment re-entrained to the shifted light cycles as exemplified in Figure 5A, while the crickets treated with double RNAi including *opLW* often lost entrainability to the shifted LDs (example, Figure 5B). A masking effect was observed again when the subjective night was exposed to light (Figure 5). The ratios of animals re-entrained to advanced or delayed LDs were shown in Figure 6A; crickets treated with *opLW* dsRNA combined with *opB* or *opUV* dsRNA lost re-entrainment in 47% and 64% in advance shifts, and 6% and 37% in delay shifts, respectively. The numbers of transient cycles in re-entrained crickets treated with ds*opLW* combined with ds*opB* or ds*opUV* were 6.4 ± 1.4 days (average ± S.D.) and 6.9 ± 1.2 days, respectively, for the advance. Those for delay shifts were 5.3 ± 3.1 days and 5.2 ± 2.6 days for RNAi with ds*opLW* and ds*opB*, and ds*opLW* and ds*opUV*, respectively. These values were not statistically significant (P > 0.05, ANOVA) but greater than those for control crickets.

## Discussion

### Opsin gene expression and RNAi

The amino acid sequences of cDNAs of the opsin genes obtained from the compound eye are identical to those reported by Henze et al. [21]. Their expression profiles in the compound eye were also reproduced and were quite similar to those found in another cricket species, *Modicogryllus siamensis* [18]. Thus the regional pattern of opsin gene expressions is conserved among cricket species and may have some biological significance.

In some insects, opsin genes are rhythmically expressed in both LD and DD conditions [23,24]. A significant daily rhythm was detected also in *G. bimaculatus*: mRNA levels of *opB* and *opLW* genes showed a slight increase after lights off, followed by a significant decrease in the midnight then by a significant increase before lights-on (Figure 1). An insignificant but similar pattern was also observed in the *opUV* expression pattern. The increase of mRNA levels during the night might be associated with an increase of rhabdom size [25], which may be reflected in an increase in sensitivity of retinal photoreceptor as measured by electroretinogram (ERG) in constant darkness [26,27]. To confirm the role of opsins in circadian sensitivity rhythm, analysis at a protein level is required in future studies.

### The role of opsins in photic entrainment

The involvement of opsins in photic entrainment of the circadian locomotor rhythm was confirmed by examining the re-entrainment to shifted LD cycles in crickets treated with dsRNA of opsin genes. When crickets were exposed to a LD cycle advanced or delayed by 6 h, the locomotor rhythm of all negative control (*DsRed2* RNAi) crickets, *opUV* RNAi, and *opB* RNAi crickets re-synchronized with newly phased light cycles (Figure 4). However, *opLW* RNAi disrupted re-entrainment in 31% and 13% of the treated crickets for advance and delay shifts, respectively. Even in re-entrained *opLW* RNAi crickets, the transient cycles necessary for re-entrainment were significantly longer in advance shifts than in control crickets (Figure 4B). RNAi of *opLW* reduced its mRNA levels without a reduction of *opUV* and *opB* mRNA levels (Figure 2A), thus the reduction of *opLW* levels may result in reduced sensitivity to only green light. Therefore, these results suggest that OpLW plays a major role in photic entrainment, mediating the photic signals necessary for entrainment of the circadian clock.

As to the retained entrainability in some of the *opLW* RNAi crickets to the shifted LD cycle (Figures 3E, 4, and 6), one may argue the involvement of other opsins, OpUV and OpB. However, considering the loss of entrainability in the rest of *opLW* RNAi crickets, it seems more likely that those crickets still had an enough amount of OpLW for re-entrainment. A small amount of *opLW* mRNA survived *opLW* RNAi might be translated in a certain amount of OpLW that is sufficient for re-entrainment.

The hypothesis that the OpLW plays a major role in photic entrainment is strengthened by the results of re-entrainment to LD cycles in crickets treated with dsRNA of two opsin genes. The locomotor activity of all *opUV* and *opB* double RNAi crickets re-synchronized to the shifted light cycles like in intact crickets, while RNAi treatment with *opLW* combined with *opB* or *opUV* prevented re-entrainment in a significant fraction of the treated crickets (Figures 5B and 6). Successfully re-entrained crickets with double RNAi including *opLW* RNAi still tended to show transient cycles longer than in intact and *DsRed2* RNAi crickets.

We have previously shown that 7% of whole ommatidia in a compound eye are enough for entrainment to a shifted LD and that the number of transient cycles are a function of number of ommatidia irrespective of the area of the compound eye [28]. Considering the fact that the *opLW* is expressed most part of the compound eye (Additional file 1: Figure S1) [21], it is thus most likely that the *opLW* photoreceptors additively provide photic information to the circadian clock to reset it: The magnitude of the phase shift probably depends on the amount of information, or amount of neurotransmitter, delivered through the *opLW* pathway. This hypothesis is supported by the fact that light entrainability depends on light intensity of the light cycle [29,30].

The crickets, *G. bimaculatus*, live in the grass fields where they are surrounded by a green light rich environment [31,32]. It is thus likely that they have evolved to use the green light for entrainment of their circadian rhythm. They may have acquired the high level of *opLW* expression and large number of retinula cells expressing *opLW* for this purpose. However, the results of present study do not exclude a possibility that OpUV and OpB play a subtle role in photic entrainment. In *D. melanogaster*, three opsins, i.e. Rh1, Rh5 and Rh6 are known to play some role as circadian photoreceptor in addition to CRY [2,9,33]. Thus a possibility still remains that the three opsins cooperatively contribute to the entrainment in *G. bimaculatus*.

Apart from photic entrainment, the crickets showed an enhanced activity when the subjective night was exposed to light by phase shifts of LD cycle (Figures 3 and 5). This positive masking effect of light is known to be induced by a pathway bypassing the circadian clock [29,34]. Since the effect was observed in all opsin RNAi crickets (Figures 3 and 5), it is likely that all opsin pathways and/or ocelli are involved in the masking. Ocelli are shown to express another type of *opLW* in *G. bimaculatus* [21], and its role should be examined in future studies.

### Cerebral photoreceptor vs compound eyes

In *D. melanogaster* and the monarch butterfly (*D. plexippus*), *cry1* is believed to be the principal photoreceptor for entrainment of the clock [2,6]. It is expressed in some of the cerebral clock neurons, activated by light, and leads to TIM degradation. In hymenopteran species, however, *tim* and *cry1* are absent in their genome [35]. In honeybees pteropsin has been postulated to play a role in photic entrainment [36]. It is expressed in some neurons in the optic lobe and cerebral lobe which are closely located to the putative clock neurons expressing PDF in the brain [7,37]. In contrast, the cricket relies on only the compound eye for photic entrainment as also known for cockroaches [38,39]. Thus, the clock receives photic information through neurotransmission via several neuronal elements. It is a challenging question why crickets and cockroaches rely solely on the compound eye. The species using *cry1* or pteropsin as the photoreceptor are restricted to holometabolous insects that lack the compound eyes during their larval stages, thus they may have to use cerebral photoreceptor for entrainment during development. It is interesting that those insects also use the compound eyes as adults [9,33]. In contrast, crickets and cockroaches are hemimetabolous insects possessing the compound eye from the first instar stage and they use it from the early developmental stage. Thus usage of cerebral photoreceptors might be later acquired.

This view makes sense given a commonality of circadian photoreceptors between crickets and mammals. In mammals, melanopsin expressed in retinal ganglion cells plays a major role in the photic entrainment of the circadian clock in the suprachiasmatic nucleus [15,16]. Melanopsin is a photoreceptor coupled with Gq type G-protein [40]. Since insect opsins expressed in retinula cells are also Gq-coupled photoreceptors, our results suggest that the insect retinal circadian photoreceptor may have common origin with the mammalian circadian photoreceptors but have diversified to receive different wavelength to adapt to their habitat.

### Two entrainment pathways of the compound eye

The compound eye is known to provide photic information also for mutual synchronization between circadian clocks in the two optic lobes. By partial destruction of the compound eye, the dorso-caudal region has been shown to play an essential role in photoreception for the coupling [41]. The photic information perceived by this area is mediated by a group of large neurons, so-called medulla bilateral neurons, which send the information to the contralateral medulla area of the optic lobe, putative locus of the circadian clock [42]. Thus, it is likely that separate pathways are used for photic entrainment and for mutual coupling of the circadian clock in the cricket. Whether common photoreceptors are used for the two entrainment pathways is to be examined in future studies.

## Conclusions

We have investigated the role of opsin genes that are expressed in the compound eye in photic entrainment of the locomotor rhythm in the cricket *G. bimaculatus*. Our analysis using RNAi technology revealed that the knocking down of *opLW* gene expression either disrupted the entrainment to free-run in LD conditions or lengthened the transient cycles for re-entrainment to LDs shifted by 6 h. However, no significant effects were observed when other two opsin genes, *opUV* and *opB*, were knocked down. The data clearly show that the green-sensitive opsin, OpLW, is the major photoreceptor for the entrainment of the cricket’s circadian clock. The retinula cells expressing *opLW* distributed almost whole compound eye except for the DRA, suggesting that the photic signals perceived by *opLW* expressing retinula cells probably contribute additively to accomplish the phase setting of the circadian clock. The present study not only extends our knowledge on the photic entrainment mechanism of hemimetabolous insects but also will promote the investigation of the mechanism at the cellular and molecular levels, which still remains largely unknown.

## Additional file

Additional file 1: Figure S1.
*In situ* hybridization of *opLW*, *opUV* and *opB* in the adult compound eye of the cricket *Gryllus bimaculatus*. Scale bar, 100 μm. DRA, dorsal rim area; ON, optic nerve; OL, optic lobe.
