# Supplementary material for: Green-sensitive opsin is the photoreceptor for photic entrainment of an insect circadian clock
Source: Zoological Lett. 2015 Feb 26;1:11. doi: 10.1186/s40851-015-0011-6 (PMC4657349; doi:10.1186/s40851-015-0011-6)
Supplement: Additional file 1: Figure S1. — In situ hybridization of opLW, opUV and opB in the adult compound eye of the cricket Gryllus bimaculatus. Scale bar, 100 μm. DRA, dorsal rim area; ON, optic nerve; OL, optic lobe. [file 40851_2015_11_MOESM1_ESM.docx]

Additional File

Figure S1. *In situ* hybridization of *opLW*, *opUV* and *opB* in the adult compound eye of the cricket *Gryllus bimaculatus*. Scale bar, 100 μm. DRA, dorsal rim area; ON, optic nerve; OL, optic lobe.
